# Indictment for Manslaughter

**Published:** 1846-10

**Authors:** 


*Indictment for Manslaughter.*—The grand jury at a recent Court session in this city presented a bill of indictment for manslaughter against a Dr. Frank, an irregular German practitioner of this city. The bill, we understand, charges him with using means to effect delivery in a case of labour, by which the woman suffered injuries so as to cause her death during, or shortly after the manipulations. We abstain from details until the case is adjudicated, and we can derive them from an authentic source.

